# Cardiac Function is not Susceptible to Moderate Disassembly of Mitochondrial Respiratory Supercomplexes

**DOI:** 10.3390/ijms21051555

**Published:** 2020-02-25

**Authors:** Xavier R. Chapa-Dubocq, Keishla M. Rodríguez-Graciani, Roberto A. Guzmán-Hernández, Sehwan Jang, Paul S. Brookes, Sabzali Javadov

**Affiliations:** 1 Department of Physiology, University of Puerto Rico School of Medicine, San Juan, PR 00936-5067, USA; xavier.chapa@upr.edu (X.R.C.-D.); keishla.rodriguez20@upr.edu (K.M.R.-G.); roberto.guzman7@upr.edu (R.A.G.-H.); sehwan.jang@upr.edu (S.J.); 2 Department of Anesthesiology, University of Rochester School of Medicine & Dentistry, Rochester, NY 14642, USA; paul_brookes@urmc.rochester.edu

**Keywords:** heart, mitochondria, respiratory chain supercomplexes, respirasome, ethanol

## Abstract

Mitochondrial respiratory chain supercomplexes (RCS), particularly, the respirasome, which contains complexes I, III, and IV, have been suggested to participate in facilitating electron transport, reducing the production of reactive oxygen species (ROS), and maintaining the structural integrity of individual electron transport chain (ETC) complexes. Disassembly of the RCS has been observed in Barth syndrome, neurodegenerative and cardiovascular diseases, diabetes mellitus, and aging. However, the physiological role of RCS in high energy-demanding tissues such as the heart remains unknown. This study elucidates the relationship between RCS assembly and cardiac function. Adult male Sprague Dawley rats underwent Langendorff retrograde perfusion in the presence and absence of ethanol, isopropanol, or rotenone (an ETC complex I inhibitor). We found that ethanol had no effects on cardiac function, whereas rotenone reduced heart contractility, which was not recovered when rotenone was excluded from the perfusion medium. Blue native polyacrylamide gel electrophoresis revealed significant reductions of respirasome levels in ethanol- or rotenone-treated groups compared to the control group. In addition, rotenone significantly increased while ethanol had no effect on mitochondrial ROS production. In isolated intact mitochondria in vitro, ethanol did not affect respirasome assembly; however, acetaldehyde, a byproduct of ethanol metabolism, induced dissociation of respirasome. Isopropanol, a secondary alcohol which was used as an alternative compound, had effects similar to ethanol on heart function, respirasome levels, and ROS production. In conclusion, ethanol and isopropanol reduced respirasome levels without any noticeable effect on cardiac parameters, and cardiac function is not susceptible to moderate reductions of RCS.

## 1. Introduction

Transport of electrons through the electron transport chain (ETC), coupled with oxidative phosphorylation, generates the proton motive force in the inner mitochondrial membrane (IMM), and thus drives ATP production by F_O_F_1_-ATP synthase. To date, at least three structural models known as the fluidity (random collision) model, the solid model, and the plasticity model have been proposed to explain the structural organization of ETC complexes in the IMM [1]. According to the fluidity model, ETC complexes act as separate units to transport electrons by free diffusion using electron carrier proteins such as cytochrome c and coenzyme Q [2]. However, studies since 2000 proposed a solid model where individual ETC complexes can assemble into supramolecular structures known as respiratory chain supercomplexes (RCS) [3]. In addition, the plasticity (hybrid) model developed recently proposes that ETC complexes can function in both forms (individually and assembled in RCS) simultaneously [4]. 

The main RCS, termed the “respirasome”, contains ETC complexes I, III, and IV in a different molar ratio [5,6]. The respirasome has been suggested to maintain the structural integrity of individual ETC complexes [7], improve the efficiency of electron channeling by preventing electron leakage [8], and reduce mitochondrial ROS production [9]. Disassembly of RCS was observed in human diseases such as Barth syndrome [10,11], neurodegenerative [12,13] and cardiovascular [14,15] diseases, diabetes [16], and aging [17]. However, the physiological role of RCS in a high energy-demanding tissue such as the heart has yet to be determined. Studies from our and other groups demonstrated that cardiac ischemia–reperfusion and heart failure are associated with disruption of the respirasome structural integrity [14,18,19]. Coronary microembolization-induced heart failure in dogs for 3 weeks reduced respirasome levels and increased the levels of individual complexes I, III, and IV, suggesting a possible cause–effect relationship between RCS dissociation and cardiac dysfunction observed in these animals [14]. Early (5 min) reperfusion after global ischemia had no effect on respirasome, and sustained reperfusion (60 min) induced only a ~5% decrease in respirasome levels despite severe cardiac dysfunction [18]. Furthermore, 2- and 28-days post-infarction remodeling did not affect the respirasome levels in female rats, despite significant reductions of ejection fraction which were 44% and 48% less, respectively, than in sham-operated animals [19]. These studies raised the question of whether RCS disassembly plays a causative role in the pathogenesis of mitochondria-mediated cardiac dysfunction during coronary heart diseases.

In this study, we examined a cause-and-effect relationship between respirasome depletion and cardiac function in rat intact hearts. Results demonstrated that the disassembly of respirasome by the ETC complex I inhibitor, rotenone, was associated with diminished cardiac function. However, ethanol and isopropanol induced respirasome disassembly without any noticeable effect on cardiac function. 

## 2. Results

### 2.1. Cardiac Function

**Figure 1:**
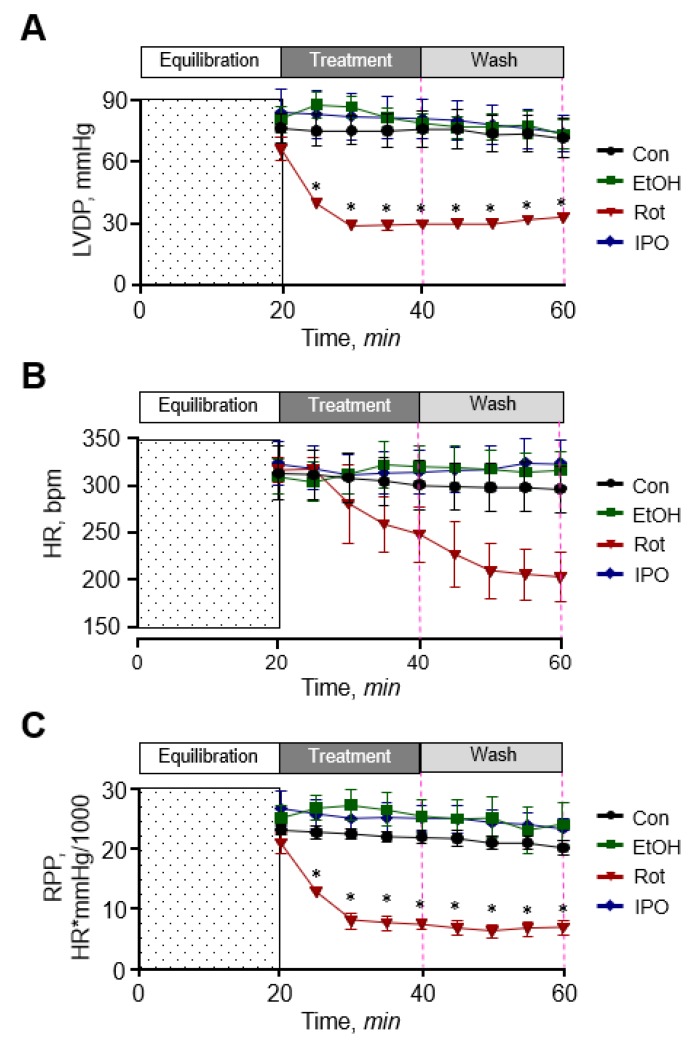
Cardiac function. (**A**) Left ventricular developed pressure (LVDP). (**B**) Heart rate (HR). (**C**) Rate pressure product (RPP). LVDP is the difference between left ventricular systolic pressure and left ventricular end-diastolic pressure. RPP was calculated as the product of LVDP and HR. Con, control; EtOH, ethanol; IPO, isopropanol; Rot, rotenone. * *p* < 0.05 vs. Con, *n* = 6–8.

First, we examined the effect of ethanol, isopropanol, and rotenone on cardiac function. As shown in Figure 1, ethanol and isopropanol had no significant effects on cardiac function as evidenced by left ventricular developed pressure (LVDP), rate pressure product (RPP), and heart rate (HR) that remained unchanged in the presence and absence of ethanol or isopropanol. The data for cardiac function parameters in these groups were similar to those in the control (non-treated) group. In contrast, rotenone significantly diminished cardiac function as evidenced by reduced LVDP (by 60%) and HR (by 32%) at the end of 20-min perfusion with the inhibitor (rotenone group). Cardiac function was not recovered when perfusion was continued with Krebs-Henseleit solution not containing rotenone (rotenone-wash group), and LVDP and HR were 58% and 39% less (*p* < 0.01 for both) than in the ethanol-wash group by the end of 40-min perfusion (Figure 1A,B). As a result, RPP, which reflects cardiac work, was 76% and 75% (*p* < 0.05) less in the presence (rotenone group) and absence of rotenone (rotenone-wash group), respectively (Figure 1C). In addition, the hearts treated with rotenone developed irregular beating patterns (arrhythmias). Thus, ethanol and isopropanol do not impair cardiac function, whereas rotenone induces a significant dysfunction of the heart.

### 2.2. Mitochondrial Respiratory Function, Mitochondrial ROS Production, and PTP Opening

**Figure 2:**
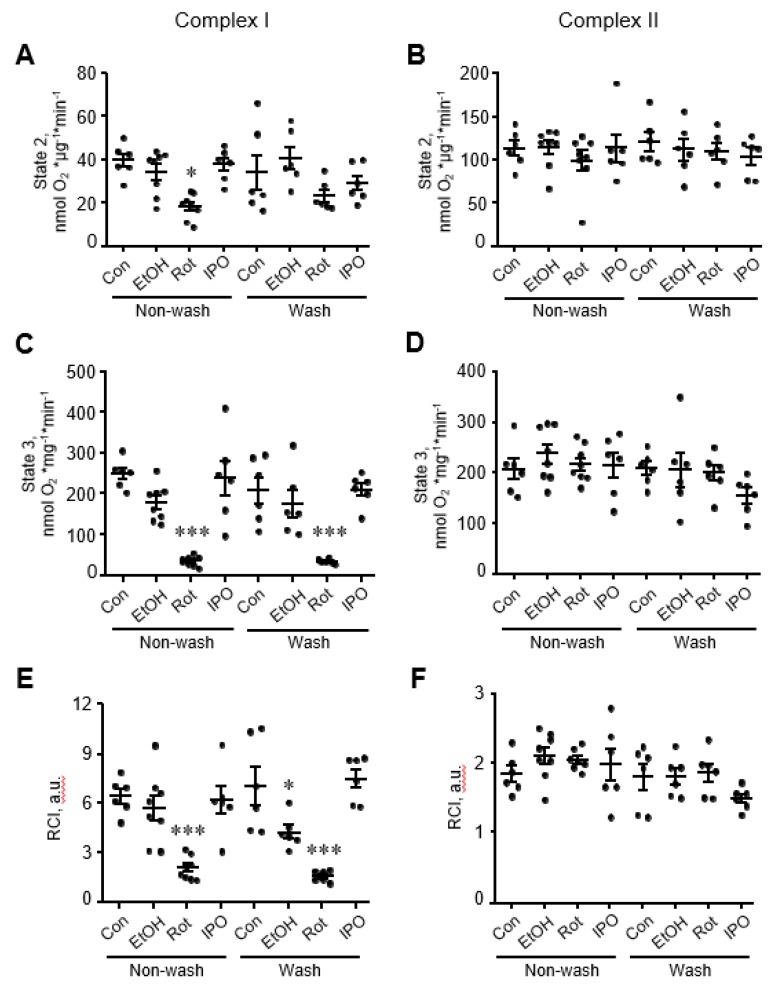
Mitochondrial respiration rates for electron transport chain (ETC) complexes I and II. State 2 (**A**,**B**), state 3 (**C**,**D**), and respiratory control index (RCI) (**E**,**F**) for complexes I and II. Mitochondrial respiration rates were measured in isolated mitochondria using substrates for complexes I (α-ketoglutarate and L-malate) and II (succinate) in the absence (state 2) or presence of ADP (state 3). Oxygen consumption rates are presented in nmol oxygen/min per mg of mitochondrial protein. RCI by Lardy was calculated as the ratio of state 3 to state 2. Con, control; EtOH, ethanol; IPO, isopropanol; Rot, rotenone. * *p* < 0.05, *** *p* < 0.001 vs. Con, *n* = 6–8 per group.

Analysis of mitochondrial respiration rates in mitochondria isolated from ethanol, isopropanol, or rotenone-treated hearts showed that rotenone reduced by 80% (*p* < 0.001 vs. control), whereas ethanol and isopropanol had no effect on state 2 and state 3 respiration rates for complexes I and II (Figure 2A-D). State 3 for complex I remained reduced in rotenone-treated hearts even when perfusion continued with Krebs-Henseleit solution containing no rotenone (wash group, Figure 2C). Likewise, RCI for complex I in rotenone-treated hearts was 65% (*p* < 0.05, Figure 2E) lower than that in control hearts and remained unchanged when rotenone was removed from the perfusion media. Interestingly, removing ethanol (wash group) from the perfusion media reduced the RCI for complex I by 48% (*p* < 0.05, Figure 2E) in ethanol-treated hearts. State 3 respiration rate and RCI for complex II were not affected in mitochondria isolated from either ethanol, isopropanol, or rotenone-treated hearts (Figure 2B,D,F).

**Figure 3:**
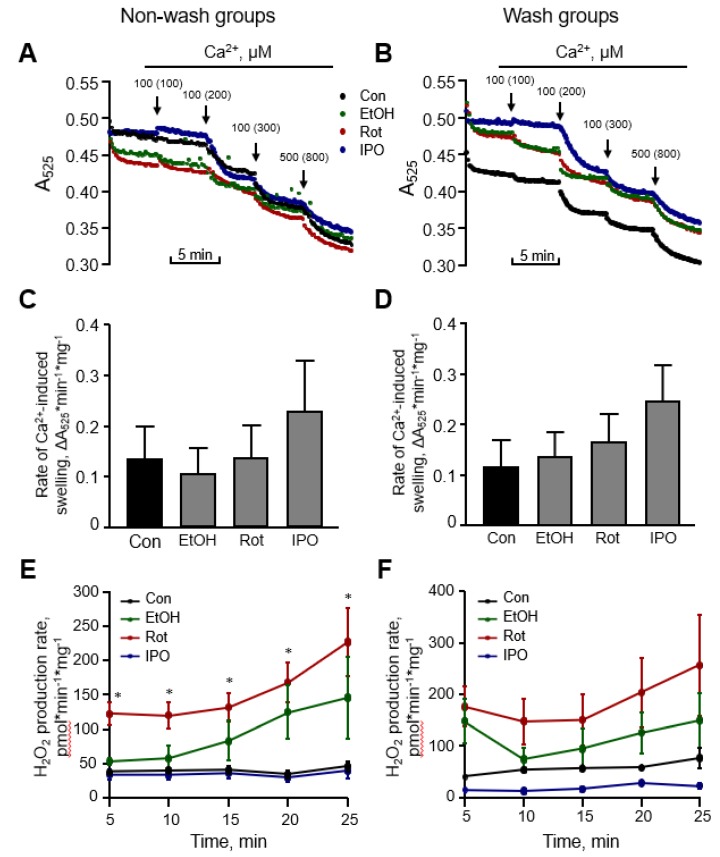
Mitochondrial swelling and reactive oxygen species (ROS) production rates. Representative curves (**A**,**B**) and quantitative data (**C**,**D**) of mitochondrial swelling, and the rates of mitochondrial ROS production rates (**E**,**F**). Mitochondrial swelling as a marker of permeability transition pore (PTP) opening was induced by addition of CaCl_2_, shown as the added and the final (cumulative) concentration in brackets, and measured by monitoring the decrease in light scattering at 525 nm. Quantitative data (**C**,**D**) are given for mitochondrial swelling rates induced by 200 µM Ca^2+^. Basal (no Ca^2+^) rates of mitochondrial ROS production (**E**,**F**) were measured for 25 min with 5-min intervals. Con, control; EtOH, ethanol; Iso, isopropanol; Rot, rotenone. * *p* < 0.05 Con vs. Rot, *n* = 6–8 per each group.

Next, we evaluated the effects of ethanol, isopropanol or rotenone on mitochondrial ROS production and mitochondrial swelling in the heart (Figure 3). Measurement of mitochondrial swelling as a marker of the PTP opening is used to determine the Ca^2+^ retention capacity of mitochondria. Analysis of mitochondria isolated from ethanol-, isopropanol- or rotenone-treated hearts demonstrated no difference in total mitochondrial swelling in the wash and non-wash groups (Figure 3C,D). Analysis of ROS with Amplex Red revealed that mitochondrial ROS production was approximately two times higher in the rotenone-treated hearts without subsequent perfusion, in comparison with the control group (Figure 3E, non-wash groups). No significant difference in mitochondrial ROS was found after removing rotenone from the perfusion medium, even though a higher trend was observed (Figure 3F, wash groups). Ethanol and isopropanol did not exert a significant effect on mitochondrial ROS production.

### 2.3. Mitochondrial Respirasome Levels

**Figure 4:**
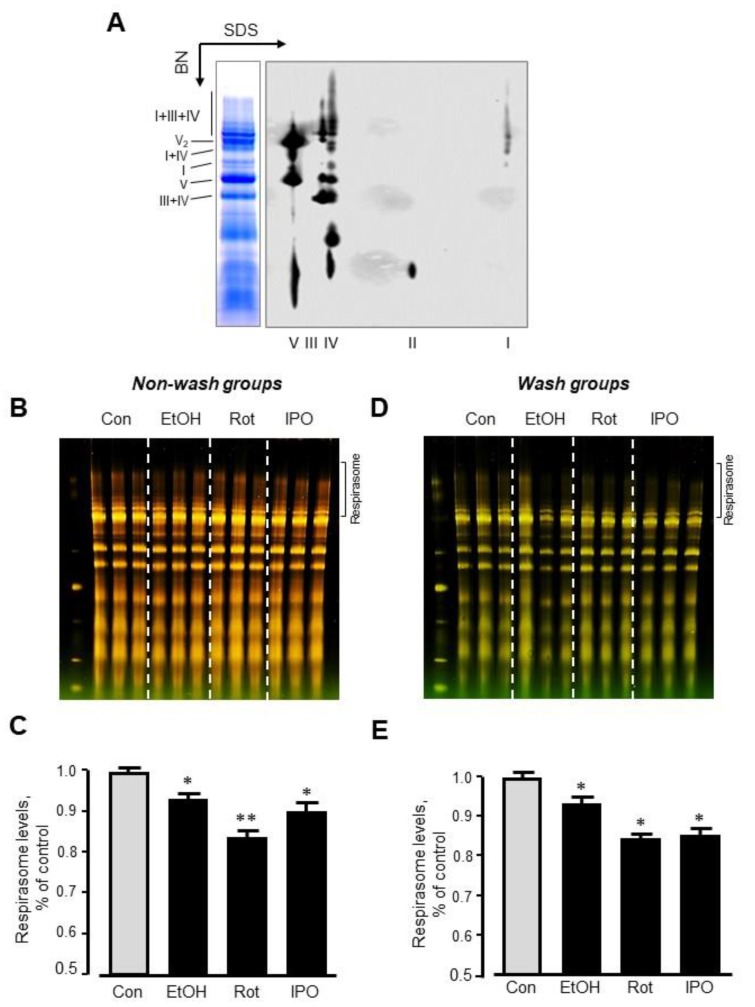
Respirasome levels in mitochondria isolated from non-wash and wash groups by blue-native polyacrylamide gel electrophoresis (BN-PAGE). (**A**) Representative two-dimensional BN-PAGE (2D BN-PAGE) of RCS. Mitochondria were treated with digitonin and subjected to 2D BN-PAGE. ETC complexes were visualized using antibodies against the subunits NDUFB8 (complex I), SDHB (complex II), UQCRC2 (complex III), MTCO1 (complex IV), and ATP5A (complex V). (**B**,**D**) Representative BN-PAGE images of RCS. (**C**,**E**) Quantitative data of RCS levels. The levels of respirasome were expressed as a percent change of control (Con). EtOH, ethanol; IPO, isopropanol; Rot, rotenone. * *p* < 0.05; ** *p* < 0.01 vs. Con, *n* = 3 per each group.

Analysis of RCS by blue-native polyacrylamide gel electrophoresis (BN-PAGE) in mitochondria isolated from ethanol-, isopropanol- or rotenone-treated hearts without subsequent perfusion (non-wash groups) revealed, respectively, a 7% (*p* < 0.05), 17% (*p* < 0.01) and 10% (*p* < 0.05) reduction of respirasome, compared to the control group (Figure 4A–C). We revealed similar results (6%, 16%, and 15% reduction for ethanol-, isopropanol- or rotenone-treated hearts, respectively, *p* < 0.05 for all) in wash groups when the hearts were perfused with normal Krebs-Henseleit solution after treatment with ethanol, isopropanol, or rotenone (Figure 4D,E). The differences in respirasome levels were consistent in both group treatments.

**Figure 5:**
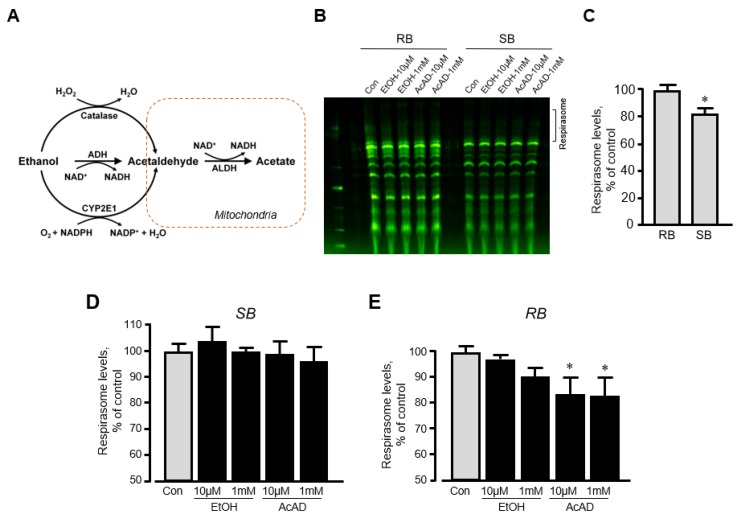
The effects of acetaldehyde (AcAD) and ethanol (EtOH) on the respirasome assembly in mitochondria *in vitro*. Mitochondria isolated from healthy (non-treated) rat hearts were diluted in either sucrose or respiration buffer and incubated with AcAD or EtOH for 60 min at 37 °C. At the end of the incubation period, RCS were analyzed by BN-PAGE. (**A**) Diagram of EtOH metabolism in the cell. (**B**) Representative images of RCS in mitochondria diluted in either sucrose or respiration buffer. (**C**) Respirasome levels in mitochondria diluted in either sucrose or respiration buffer. (**D**,**E**) The effects of AcAD and EtOH at high (1 mM) and low (10 µM) concentrations on respirasome levels in mitochondria diluted in either sucrose (**D**) or respiration buffer (**E**). Respirasome levels were presented as the percentage of control (Con). * *p* < 0.05 vs. Con, *n* = 3 per each group. RB: Respiration buffer, SB: Sucrose buffer.

We have previously shown that in vitro incubation of isolated mitochondria with rotenone induced disassembly of RCS and reduced the respirasome level by 13% [20]. To clarify whether ethanol can exert a similar direct effect in vitro on respirasome assembly, we incubated mitochondria isolated from the intact rat hearts with either ethanol or acetaldehyde. We assessed the effect of acetaldehyde based on the fact that (1) ethanol is converted into acetaldehyde by alcohol dehydrogenase in the cell (Figure 5A), and (2) acetaldehyde has detrimental effects on mitochondrial respiration [21]. Mitochondria resuspended in two different buffers (sucrose and respiration) were used in these in vitro experiments (see *Materials and Methods*). The respiration buffer which contained α-ketoglutarate and L-malate was used to test whether in vitro changes in RCS assembly require an active ETC. Results showed that incubation of mitochondria in two buffers differently affects RCS assembly; incubation in the sucrose buffer stimulated RCS disassembly (17% reduction, *p* < 0.05) suggesting the importance of an active ETC for the structural integrity of respirasome (Figure 5B,C). Ethanol at either low (10 μM) or high (1 mM) concentration had no effect on RCS assembly in both buffers (Figure 5D,E). However, in the respiration buffer but not sucrose buffer, acetaldehyde was able to reduce RCS levels by 16% (*p* < 0.05 vs. control for both) at either low or high concentration (Figure 5E). These data suggest that active ETC is required for the maintenance of the structural organization of RCS assembly.

## 3. Discussion

In this study, for the first time, we provide evidence that the cardiac function of isolated Langendorff-perfused rat hearts is not susceptible to moderate disassembly of mitochondrial RCS. Particularly, we demonstrated that: (1) primary (ethanol) and secondary (isopropanol) alcohols decrease mitochondrial respirasome levels without any effect on cardiac function; (2) inhibition of complex I with rotenone stimulates disassembly of respirasome associated with diminished cardiac function; (3) ethanol and isopropanol had no effect on mitochondrial respiration (state 3) whereas rotenone markedly reduced it; and (4) ethanol had no direct effects on respirasome assembly, but its metabolite acetaldehyde stimulated dissociation of RCS in mitochondria in vitro with active ETC.

A number of studies elucidated an associative link between RCS disassembly and cardiac dysfunction caused by ischemia–reperfusion injury [15,19], heart failure [14,22] and aging [23]. Furthermore, defective cardiolipin remodeling due to tafazzin deficiency in patients with Barth syndrome [10] and in early embryonal tafazzin knockdown mice [11] diminished heart function and caused respirasome disassembly associated with the reduced enzymatic activity of individual ETC complexes. However, several studies challenged a causative role of RCS disassembly in cardiac/mitochondrial dysfunction. We have previously shown that cardiac mitochondria isolated from tafazzin knockdown mice demonstrated a 40% reduction of respirasome levels; however, basal mitochondrial ROS levels were similar to wild-type counterparts [15]. Interestingly, ex-vivo ischemia-reperfusion in rat hearts did not affect respirasome assembly at early (5 min) reperfusion, and induced only a ~5% reduction of respirasome levels after 60 min reperfusion despite severe mitochondria alterations (high ROS production and PTP opening) and very low post-ischemic recovery (23% of pre-ischemia) of the heart [15]. In addition, in vivo myocardial infarction with or without subsequent reperfusion for either 2 or 28 days had no negative effect on the structural integrity of the respirasome [19]. Tafazzin deficiency in mice initiated at an adult age did not affect mitochondrial respiration and oxidative activity in the heart despite a 40% reduction of cardiolipin [24], a mitochondrial signature phospholipid which has been shown to play an important role in the maintenance of the structural integrity of ETC complexes [25] and RCS [26]. Furthermore, the hearts of tafazzin knockdown mice exhibited similar sensitivity as wild-type mice to *ex-vivo* ischemia-reperfusion as evidenced by the lack of differences in the infarction size and post-ischemic LVDP recovery [24]. In a recent study, liver damage induced by a high cholesterol diet was associated with mitochondrial alterations and RCS disassembly. However, treatment with glutathione ethyl ester significantly attenuated liver damage and recovered mitochondrial respiration with no effects on RCS levels [27]. Interestingly, in vitro studies demonstrated that respirasome does not enhance electron channeling as the electron carrier molecules, quinone and quinol, diffuse freely across the RCS [28]. This conclusion was substantiated by a recent study when CoQ trapping in the RCS I+III_2_ in vitro reduced complex I turnover [29], suggesting that substrate channeling does not support or facilitate respiration. However, previous experiments performed on mtDNA mutant cybrid cell lines demonstrated the threshold between mitochondrial RCS disassembly and respiration defects [30]. These studies suggest that the extent of RCS disassembly should reach a threshold level to provoke cardiac dysfunction. Hence, moderate disintegration of RCS induced by ethanol and isopropanol might not be sufficient to alter mitochondrial bioenergetics and thus, affect heart contractility. Indeed, mitochondrial respiration (state 3 and RCI) was not markedly affected in alcohol-treated hearts (Figure 2).

We discovered the capacity of ethanol to stimulate disassembly of respirasome unexpectedly, in experiments elucidating the effect of ethanol as a vehicle for rotenone on the respirasome integrity. Indeed, an associative link exists between sustained ethanol consumption and heart diseases [31], and ethanol has been shown to exert detrimental effects on mitochondria; it diminishes mitochondrial respiration, increases mitochondrial ROS production [32,33], and induces mitochondrial DNA damage [34]. Moreover, ethanol injected to mice significantly reduced the activity of ETC complexes and increased mitochondrial ROS production in the brain cortex [35,36]; however, the effects of ethanol on RCS assembly were not assessed previously. Interestingly, we observed similar effects for isopropanol on RCS levels and heart function. The effect of alcohol (ethanol and isopropanol) to induce RCS disassembly with unaltered cardiac function indicates that heart contractility is independent of the supramolecular structural organization of ETC complexes. In addition, ethanol-induced disassembly of RCS did not stimulate mitochondrial ROS production (Figure 3E,F). The results of our study provide an in vivo argument against the possible role of RCS in electron channeling. This conclusion is consistent with previous in vitro studies [28,29] that suggested a lack of electron channeling in respirasome. Our findings suggest that RCS disassembly (at least up to 17%, as shown in the present study) does not induce cardiac dysfunction, and RCS disassembly can occur as a consequence rather than a cause of ischemia-reperfusion injury, diabetes, and other diseases. On the other hand, the complete disassembly of RCS was not induced under these conditions, which could allow the majority of intact RCS to satisfy energy demands through a compensatory mechanism of cardiac cells. The mechanisms underlying the effect of ethanol on respirasome assembly are not clear and require further studies. Incubation with ethanol did not affect respirasome assembly in isolated mitochondria in vitro (Figure 5D,E) suggesting that the effect to induce RCS disassembly can be mediated through indirect mechanisms.

In the cytoplasm, alcohol dehydrogenase converts ethanol into acetaldehyde which is metabolized in the mitochondria by aldehyde dehydrogenase [37]. Acetaldehyde accumulation and activation of aldehyde dehydrogenase 2, a mitochondrial isozyme of aldehyde dehydrogenase, have been shown to diminish mitochondrial respiration [38]. Our data demonstrated that acetaldehyde reduces respirasome levels in mitochondria incubated in respiration buffer (Figure 5D,E), suggesting that active ETC is necessary for acetaldehyde to disrupt the respirasome. Furthermore, ethanol has been shown to alter mitochondrial dynamics through activation of mitochondrial translocation of Drp1 and proteolytic cleavage of L-OPA1 to S-OPA1 [33]. The fusion protein L-OPA1 is mostly localized in the IMM and plays a key role in the maintenance of the cristae shape and RCS integrity [39]. 

The molecular role of the mitochondrial RCS, particularly respirasome, is still under question. As mentioned above, respirasome is thought to reduce mitochondrial ROS generation [9], improve the efficiency of electron transport [8], and maintain the structural integrity of individual ETC complexes such as complex I [7]. Nevertheless, two recent studies have found that the respirasome is not kinetically important for channeling electron across the ETC [28,40]. These studies suggest that RCS may be an evolutionary adaptation to a heavily populated protein environment to avoid protein aggregation. On the contrary, a study using flux control analysis found evidence that respirasome does provide a kinetic advantage in electron channeling [41,42]. Interestingly, in this study, we found that only ethanol wash groups had a lower RCI compared to control while the other groups remained unaffected, suggesting that the respirasome may not alter electron channeling. Furthermore, cardiac function was unaffected by ethanol, suggesting that ATP demands were satisfied. 

Our study, similar to other studies, encompasses certain limitations: (1) we did not measure ATP levels and evaluate the contribution of glycolysis to ATP production, which can compensate for ATP demands during cardiac dysfunction [43]; (2) we assessed the effects of all three compounds during a short period of perfusion (1 h max perfusion time); and (3) the hearts were only perfused with a glucose-based solution. Other substrates (e.g. free fatty acids) in the perfusion media might differently affect the respirasome assembly. It should be noted that inhibition of complex I by rotenone could aggravate cardiac function due to blockage of electron transfer and high ROS production independent of moderate respirasome disassembly. In addition, certain challenges associated with analysis of RCS by the BN-PAGE technique should be taken into consideration [44]. Apparently, disadvantages of the technique including, among others, solubilization of RCS and isolation of mitochondria can affect the native structure of respirasome, and compromise understanding its role in mitochondrial bioenergetics and undermine the contribution of RCS disassembly to cardiac function.

In conclusion, this study, for the first time, provides evidence that that cardiac function from Langendorff-perfused rat hearts is not susceptible to moderate disassembly of mitochondrial RCS. We observed that ethanol and isopropanol can stimulate disassembly of the respirasome without significant effects on mitochondrial respiration. Likely, severe disassembly of the respirasome beyond a critical threshold can diminish mitochondrial bioenergetics and cardiac function. Based on our findings, we suggest that ETC complexes can exist either individually or organized into the respirasome, described as a plasticity model [4]. Dynamic interconversion between these two states (fluidity and solid) can prevent aggregation of IMM protein and maintain the structural organization of individual ETC complexes. 

## 4. Materials and Methods

### 4.1. Animals 

Three-month-old adult Sprague Dawley male rats (275–325 g) were purchased from Taconic (Hillside, NJ, USA). All experiments were performed according to protocols approved by the UPR Medical Sciences Campus Institutional Animal Care and Use Committee (approval code: A762020117) and conformed to the National Research Council Guide for the Care and Use of Laboratory Animals published by the US National Institutes of Health (2011, eighth edition).

### 4.2. Langendorff-Mode Heart Perfusion 

**Figure 6:**
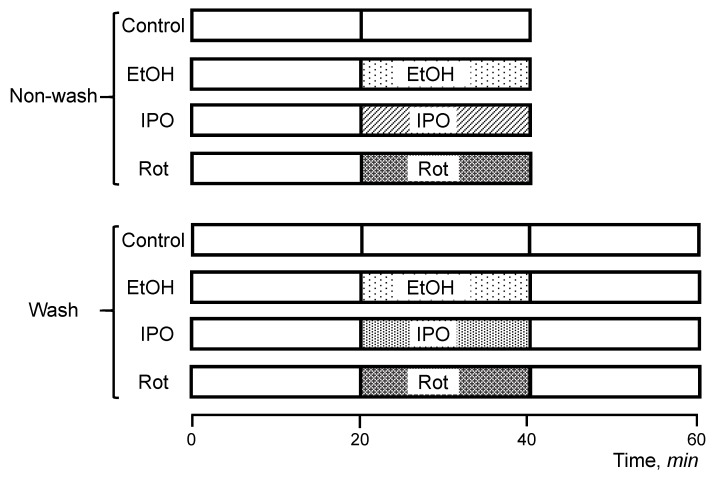
Scheme of experimental design. Rats were chosen randomly to participate in one of the following eight experimental groups: (1) control (no-treated group), 40-min perfusion (*n* = 6), (2) ethanol (EtOH) group, 20-min perfusion with EtOH (*n* = 8), (3) rotenone (Rot) group, 20-min perfusion with Rot (*n* = 8), (4) isopropanol (IPO) group, 20-min infusion with IPO (*n* = 6), (5) control (no-treated group), 60-min perfusion (*n* = 6), (6) EtOH-wash group, 20-min perfusion with EtOH followed by a 20-min perfusion without EtOH (*n* = 6), (7) Rot-wash group, 20-min perfusion with Rot followed by a 20-min perfusion without Rot (*n* = 6), and (8) IPO-wash group, 20-min perfusion with IPO followed by a 20-min perfusion without IPO (*n* = 6). Details are given in *Materials and Methods*.

Hearts were isolated and perfused according to the Langendorff-mode technique as described previously [15]. Briefly, rats were anesthetized with the anesthetic cocktail (in mg/kg body weight: 4.2 xylazine, 87.5 ketamine, and 87.5 acepromazine) administered intraperitoneally. The heart was rapidly excised, connected to the Langendorff-perfusion setup and perfused at a constant flow (10–12 mL/min per gram heart weight) with Krebs-Henseleit solution containing (in mM): 1.2 KH_2_PO_4_, 1.2 MgSO_4_, 1.2 CaCl_2_, 4.8 KCl, 118 NaCl, 25 NaHCO_3_, and 11 glucose. The buffer was equilibrated at 95% O_2_ and 5% CO_2_, pH 7.4, at 37 °C. All hearts were perfused for 20 min to stabilize cardiac function (equilibration period), and then randomly assigned to the following two sets (non-wash and wash) with 4 groups in each (Figure 6). The first set (non-wash) included the (1) control (non-treated group), 40-min perfusion (*n* = 6), (2) ethanol group, 20-min perfusion with ethanol (*n* = 8), (3) isopropanol group, 20 min perfusion with isopropanol (*n* = 6), and (4) rotenone group, 20-min perfusion with rotenone (*n* = 8). The second set (wash) included (1) control (non-treated group), 60-min perfusion (*n* = 6), (2) ethanol-wash group, 20-min perfusion with ethanol followed by a 20-min perfusion without ethanol (*n* = 6), (3) isopropanol-wash group, 20-min perfusion with isopropanol followed by a 20-min perfusion without isopropanol (*n* = 6), and (4) rotenone-wash group, 20-min perfusion with rotenone followed by a 20-min perfusion without rotenone (*n* = 6). In the first set of experiments (non-wash), the hearts were perfused with ethanol, isopropanol, or rotenone without subsequent perfusion, whereas the hearts in the second set (wash) were perfused with normal Krebs-Henseleit solution for 20 min after treatment with ethanol, isopropanol, or rotenone. The wash group was involved to verify if the effect of RCS disassembly could be reversed in the alcohol groups, considering that rotenone binding is irreversible. Ethanol and isopropanol were used at a final concentration of 1 mM. Rotenone (final concentration 0.5 µM) was dissolved in ethanol (final concentration 1 mM). Isopropanol, a secondary alcohol, was used as an alternative compound to reassess the effects of an alcohol functional group with an alternative carbon chain in the heart and clarify the specificity of the effects observed. All chemicals were handled with the appropriate personal protective equipment in accordance with the regulations established by the Biosafety and Biosecurity Committee of the UPR Medical Sciences Campus.

To examine cardiac function, a water-filled balloon inserted to the left ventricle was connected to a pressure transducer. Functional parameters including HR, the rate of contraction and relaxation (± dP/dt), aortic pressure (AP), left ventricular systolic pressure (LVSP), and left ventricular end-diastolic pressure (LVEDP) were continuously monitored using the Labscribe2 Data Acquisition Software (iWorx 308T, Dover, NH, USA). LVDP was calculated as the difference between LVSP and LVEDP (LVDP = LVSP − LVEDP). RPP was calculated as the product of LVDP and HR (RPP = LVDP × HR) to estimate cardiac work. 

### 4.3. Isolation of Mitochondria 

The isolation of mitochondria was adopted and modified from previous studies [18]. Briefly, heart ventricles were cut and homogenized using a Polytron homogenizer in ice-cold sucrose buffer containing in mM: 300 sucrose, 20 Tris-HCl, and 2 EGTA, pH 7.2, and supplemented with 0.05% BSA. The heart homogenate was centrifuged at 2000× *g* for 3 min to remove cell debris. The supernatant was centrifuged at 10,000× *g* for 6 min to precipitate mitochondria and then washed again under the same conditions in sucrose buffer (BSA-free). The final pellet containing mitochondria was resuspended in 300 µL of sucrose buffer.

### 4.4. Mitochondrial Permeability Transition Pore (PTP) Opening 

The swelling of mitochondria as an indicator of PTP opening in the presence or absence of Ca^2+^ was determined by monitoring the decrease in light scattering at 525 nm as previously described [18] with minor modifications. The swelling buffer contained in mM: 125 KCl, 20 Tris base, 2 KH_2_PO_4_, 1 MgCl_2_, 1 EGTA, 5 α-ketoglutarate, 5 L-malate, pH 7.1. Mitochondrial swelling was measured via spectrophotometry by exposing mitochondria to different concentrations of Ca^2+^ (100, 200, 300, and 800 µM) in 5-min intervals at 37 °C. Results were presented as absorbance value (A_525_) and the rate of swelling at 200 µM of Ca^2+^ (ΔA_525_·min^–1^·mg^−1^).

### 4.5. Mitochondrial ROS Production 

To measure mitochondrial ROS production, H_2_O_2_ levels were examined in isolated mitochondrial suspension using Amplex Red (Molecular Probes, Eugene, OR, United States, final concentration 50 µM). The fluorescence intensity of Amplex Red was determined at an excitation of 560 nm and an emission of 590 nm. Results were expressed as in H_2_O_2_ production rate (pmol·min^−1^·mg^−1^)

### 4.6. Mitochondrial Respiration Rates

Measurement of mitochondrial respiration was performed at 37 °C using a YSI Oxygraph (Yellow Springs, OH, USA) model 5300 equipped with a Clark-type oxygen electrode. Oxygen consumption rates were recorded and analyzed using Chart5 (Powerlab, Colorado Springs, CO, USA). Mitochondria were suspended in a buffer containing in mM: 125 KCl, 20 MOPS, 10 Tris base, 0.5 EGTA, and 2 KH_2_PO_4_, pH 7.2, supplemented with either of the following substrates to measure complex I- and complex II-mediated respiration rates, respectively: (1) 2.5 mM α-ketoglutarate and 1 mM L-malate or (2) 2.5 mM succinate and 1 µM rotenone. Respiration rates were measured in the absence (state 2) and presence (state 3) of 1 mM ADP. At the end of each run, 0.5 µM antimycin A followed by 10 mM ascorbate and 0.3 mM N, N, N′, N′-tetramethyl-p-phenylenediamine (TMPD) were added, and the new rate of respiration was measured. The O_2_ Consumption was calculated in the absence (state 2) or presence of ADP (state 3), and the respiratory control index (RCI) by Lardy was calculated as the ratio of state 3 to state 2.

### 4.7. Mitochondrial RCS Levels 

Mitochondrial RCS were analyzed by BN-PAGE as described previously [20]. Briefly, 40 μg of mitochondrial protein were dissolved in 60 μL of solubilization buffer which included: 50 mM NaCl, 50 mM imidazole-HCl, 2 mM 6-aminohexanoic acid, 1 mM EDTA, 4 μL of 20% digitonin, 1 μL protease and phosphatase inhibitor cocktails (Sigma-Aldrich, St. Louis, MO, USA), and 25 U of Benzonase^®^. BN-PAGE samples were incubated on ice for 30 min and vortexed for 5 s every 5 min. Then, the samples were centrifuged for 20 min at 20,000 × g and the supernatant was collected and mixed with 30 μL of sample buffer (50 mM NaCl, 10% glycerol, 0.001% Ponceau S, 50 mM Tris-HCl, pH 7.2). Gels were stained by Coomassie Brilliant Blue G250 and then scanned with the Odyssey CLx Infrared Imaging System (LI-COR Biosciences, Lincoln, NE, USA) at 0.5 mm focal depth and 300 ppi (pixels per inch) resolution in high-quality mode. The resulting images were analyzed with LI-COR Image Studios Lite version 5.2.5.

To examine direct effects of ethanol and acetaldehyde on mitochondria *in vitro*, mitochondria isolated from the intact heart were incubated in sucrose buffer (in mM: 300 sucrose, 20 Tris-HCl, and 2 EGTA, pH 7.2) or respiration buffer (in mM: 125 KCl, 20 Tris base, 2 KH_2_PO_4_, 1 MgCl_2_, 1 EGTA, 5 α-ketoglutarate, 5 L-malate, pH 7.1). The mitochondria in each portion were incubated in the following 5 groups: (1) control (no treatment), (2) 10 µM ethanol, (3) 1 mM ethanol, (4) 10 µM acetaldehyde, and (5) 1 mM acetaldehyde.

### 4.8. Statistical Analysis 

Data values are presented as mean ± SEM. Data were analyzed using one-way, two-way, and repeated measures ANOVA on Graphpad prism. *p* < 0.05 was considered statistically significant. In this study, the number of biological samples but not technical replicates was used as a sample size.
